# Supplementary material for: Drosophila TRIM32 cooperates with glycolytic enzymes to promote cell growth
Source: eLife. 2020 Mar 30;9:e52358. doi: 10.7554/eLife.52358 (PMC7105379; doi:10.7554/eLife.52358)
Supplement: Figure 1—source data 1. [file elife-52358-fig1-data1.docx]

**Figure 1-source data file 1**

**X-ray Diffraction Data Collection and Refinement Statistics**

|  | TRIM32-C |
| --- | --- |
| **Data Collection** |  |
| Space group | *P* 6 _5_ |
| Cell dimensions |  |
| *a*, *b*, *c* (Å) | 132.73 132.73 49.70 |
| α, β, γ (°) | 90.00 90.00 120.00 |
| Resolution (Å) | 43.45-2.60 (2.69-2.60)* |
| *R*_merge_ | 0.128 (0.680) |
| *I* / σ*I* | 14.4 (1.8) |
| Completeness (%) | 99.0 (91.9) |
| Redundancy | 7.2 (4.0) |
|  |  |
| **Refinement** |  |
| Resolution (Å) | 43.45-2.60 |
| No. reflections | 15,474 |
| *R*_work_ / *R*_free_ | 18.6/21.9 |
| No. atoms |  |
| Protein | 2258 |
| Ligand/ion | 6 |
| Water | 85 |
| *B*-factors |  |
| Protein | 37.86 |
| Ligand/ion | 26.70 |
| Water | 36.53 |
| R.m.s. deviations |  |
| Bond lengths (Å) | 0.014 |
| Bond angles (°) | 1.502 |

*Values in parentheses are for the highest-resolution shell.
