# Supplementary material for: Drosophila TRIM32 cooperates with glycolytic enzymes to promote cell growth
Source: eLife. 2020 Mar 30;9:e52358. doi: 10.7554/eLife.52358 (PMC7105379; doi:10.7554/eLife.52358)
Supplement: Figure 1—source data 2. [file elife-52358-fig1-data2.docx]

**Figure 1-source data file 2**

**Proteins identified via MS that co-purify with TRIM32-NHL**

**Experiment #1 (1418) Experiment #2 (1426.1) Experiment #3 (1426.2)**

|  | Control^1^ | TRIM32_NHL^1^ | FC^2^ | T-test  (p-value) | Control^1^ | TRIM32_NHL^1^ | FC^2^ | T-test  (p-value) | Control^1^ | TRIM32_NHL^1^ | FC^2^ | T-test  (p-value) |
| --- | --- | --- | --- | --- | --- | --- | --- | --- | --- | --- | --- | --- |
| **Bait** |  |  |  |  |  |  |  |  |  |  |  |  |
| *Drosophila* TRIM32 | 0.0 | 28.6 | INF^3^ | <0.0001 | 0.0 | 28.0 | INF | <0.0001 | 0.0 | 14.6 | INF | 0.00054 |
|  |  |  |  |  |  |  |  |  |  |  |  |  |
| **Muscle proteins** |  |  |  |  |  |  |  |  |  |  |  |  |
| Tropomyosin-1 (Tm1) | 2.0 | 55.6 | 27.6 | <0.0001 | 12.6 | 42.0 | 3.3 | 0.00013 | 0.0 | 0.0 | N/A | N/A |
| Tropomyosin-2 (Tm2) | 5.0 | 36.6 | 7.3 | <0.0001 | 13.6 | 56.0 | 4.1 | <0.0001 | 12.3 | 51.0 | 4.1 | <0.0001 |
| Troponin C (TpnC73F) | 0.0 | 13.3 | INF | 0.00027 | 0.0 | 17.3 | INF | <0.0001 | 0.0 | 15.6 | INF | <0.0001 |
|  |  |  |  |  |  |  |  |  |  |  |  |  |
| **Glycolytic enzymes** |  |  |  |  |  |  |  |  |  |  |  |  |
| Aldolase | 18.6 | 44.0 | 2.4 | 0.0013 | 0.0 | 14.6 | INF | <0.0001 | 0.0 | 7.6 | INF | 0.00033 |
| Phosphoglycerate kinase | 0.0 | 13.3 | INF | 0.00024 | 0.0 | 0.0 | N/A | N/A | 0.0 | 7.0 | INF | 0.00027 |
| Pglym78 | 0.0 | 6.0 | 6.0 | 0.018 | 0.0 | 4.6 | INF | 0.0061 | 0.0 | 8.0 | INF | 0.00071 |
| GAPDH | 20.6 | 29.0 | 1.4 | 0.02 | 0.0 | 5.6 | INF | 0.0061 | 19.0 | 24.6 | 1.3 | 0.038 |
| Pyruvate kinase | 12.3 | 27.0 | 2.2 | 0.00015 | 0.0 | 0.0 | N/A | N/A | 4.0 | 18.6 | 3.8 | 0.0015 |

^1^ Normalized total spectra

^2^ Fold change (ratio of raw spectra in TRIM32_NHL pulldown/control pulldown)

^3^ INF, Infinite (not detected in control pulldowns)
